# Gastrin-releasing peptide induces fibrotic response in MRC5s and proliferation in A549s

**DOI:** 10.1186/s12964-020-00585-y

**Published:** 2020-06-18

**Authors:** Ozgecan Kayalar, Fusun Oztay, Hurrem Gul Ongen

**Affiliations:** 1 Department of Biology, Science Faculty, Istanbul University, 34134 Vezneciler, Istanbul, Turkey; 2 Koc University Research Centre for Translational Medicine (KUTTAM), Koc University, School of Medicine, Topkapi, Davutpasa Cad. No:4 Zeytinburnu, Istanbul, Turkey; 3 Department of Pulmonary Diseases, Cerrahpasa School of Medicine, Istanbul University Cerrahpasa, Fatih, Istanbul, Turkey

**Keywords:** GRP, TGF-β signalling, Wnt signalling, Pulmonary fibrosis, MRC5 cells and A549 cells

## Abstract

**Abstract:**

Idiopathic pulmonary fibrosis (IPF) is a complex lung disease, whose build-up scar tissue is induced by several molecules. Gastrin-releasing peptide (GRP) is released from pulmonary neuroendocrine cells, alveolar macrophages, and some nerve endings in the lung. A possible role of GRP in IPF is unclear. We aimed to investigate the fibrotic response to GRP, at the cellular level in MRC5 and A549 cell lines. The proliferative and fibrotic effects of GRP on these cells were evaluated by using BrdU, immunoblotting, immunofluorescence and qRT-PCR for molecules associated with myofibroblast differentiation, TGF-β and Wnt signalling. All doses of GRP increased the amount of BrdU incorporation in A549 cells. In contrast, the amount of BrdU increased in MRC5 cells in the first 24 h, though progressively decreased by 72 h. GRP did not stimulate epithelial-mesenchymal transition in A549 cells, rather, it stimulated the differentiation of MRC5 cells into myofibroblasts. Furthermore, GRP induced gene and protein expressions of p-Smad2/3 and Smad4, and reduced the levels of Smad7 in MRC5 cells. In addition, GRP decreased Wnt5a protein levels and stimulated β-catenin activation by increasing Wnt4, Wnt7a and β-catenin protein levels. GRP caused myofibroblast differentiation by inducing TGF-βand Wnt pathways via paracrine and autocrine signalling in MRC5 cells. In conclusion, GRP may lead to pulmonary fibrosis due to its proliferative and fibrotic effects on lung fibroblasts. The abrogation of GRP-mediated signal activation might be considered as a treatment modality for fibrotic lung diseases.

Video Abstract.

**Graphical abstract:**

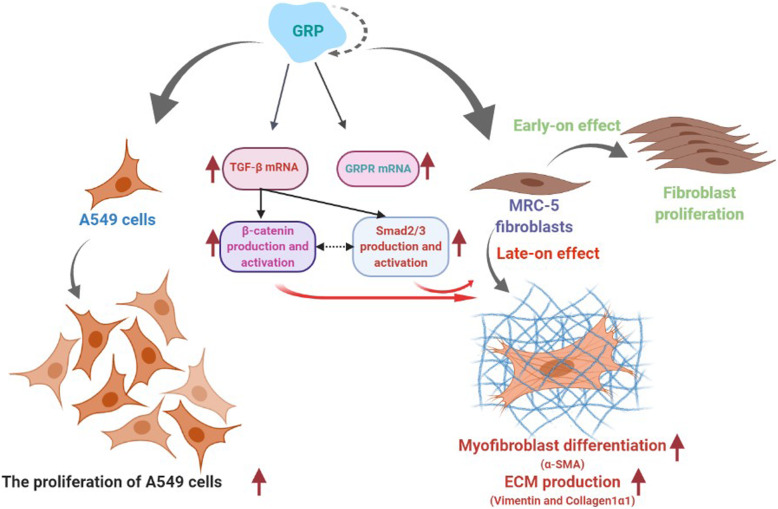

## Background

Several growth factors, cytokines, and chemokines released from pulmonary epithelial, mesenchymal and inflammatory cells contribute to the generation of pulmonary fibrosis, by regulating the proliferation and differentiation of fibroblast/myofibroblast in addition to the production, secretion, and accumulation of extracellular matrix (ECM) components [1, 2]. Although there are many molecular mechanisms involved in the pathogenesis of idiopathic pulmonary fibrosis, the fact that the fibrosis cannot be regressed completely shows us that new pathogenic mechanisms may exist under the disease.

Pulmonary neuroendocrine cells (PNECs) are found in the airway epithelium of lungs [3, 4]. They first differentiate during lung development [5]. PNECs are localized solitary or in clusters among the other epithelial cells, such as ciliated cells, goblet cells and pneumocytes. They are capable of synthesizing and releasing serotonin and peptide hormones such as bombesin, substance P, cholecystokinin, calcitonin and somatostatin regulating the biology of target cells in the lung epithelium and parenchyma by paracrine or endocrine pathways [6–8]. Thus, PNECs are effective on lung development, growth, repair and normal physiology [9–11]. Additionally, it has been demonstrated that PNECs undergo hyperplasia and/or hypertrophy in chronic obstructive pulmonary disease (COPD), asthma, bronchopulmonary dysplasia, usual interstitial pneumonia, and non-specific interstitial pneumonia [12, 13]. It is known that gastrin-releasing peptide (GRP, the mammalian homolog of the amphibian bombesin) could stimulate the proliferation of mesenchymal cells in the foetal monkey lung [14]. Ashour and colleagues detected exogenous GRP-induced myofibroblast proliferation, and an increased thickness of the alveolar wall in new-born murine lungs [15]. Recently, an increased number of PNECs and macrophages containing GRP in the lung of mouse exposed to ionizing irradiation has been reported in addition to increased alpha-smooth muscle actin (α-SMA) and p-Smad2/3 immunoreactive cells associated with the fibrotic response [16]. These data clearly show the possible contribution of GRP released from PNECs to pulmonary fibrosis generation. However, the molecular mechanisms of GRP-mediated fibrosis in the lungs are yet to be elucidated.

Transforming growth factor-beta (TGF-β) and Wnt pathways are mainly involved in the development of pulmonary fibrosis. They regulate the differentiation of fibroblast/myofibroblast as well as the production and accumulation of ECM components. In addition, TGF-β and Wnt signalling stimulates the transition of type 2 alveolar epithelial cells (AEC2) into myofibroblasts through the epithelial-mesenchymal transition (EMT) process [17]. The present study is designed to explain the molecular mechanisms of GRP-mediated fibrotic response in human lung fibroblasts (MRC5) and adenocarcinoma cell line (A549). The proliferative response, changes in the expressions of genes and proteins related with myofibroblast differentiation (α-SMA), EMT (E-cadherin), TGF-β signalling (Smad2, − 3, − 4, and − 7), Wnt signalling (Dickkopf-related protein 1 (Dkk1), Wnt4, Wnt5a, Wnt7a and β-catenin) and several proteins of ECM (collagen-1α1 and fibronectin) were analysed by quantitative polymerase chain reaction (qRT-PCR), Western blotting, and immunofluorescence labelling in MRC5 and A549 cells treated with GRP.

## Materials and methods

### Reagents, antibodies and kits

GRP, 3-(4,5-dimethylthiazol-2-yl)-2,5-diphenyltetrazolium bromide (MTT) and bovine serum albumin (BSA) were purchased from Sigma. Cell culture reagents were obtained from Gibco. Antibodies against Smad2/3, p-Smad2/3, Smad4, Wnt4a, β-catenin, Dickkopf-related protein 1 (Dkk-1), E-cadherin and glyceraldehyde 3-phosphate dehydrogenase (GAPDH) were from Santa Cruz. Antibodies against Smad7, Wnt7a, collagen 1α1, rabbit IgG, mouse IgG were from Abcam. Antibodies against Wnt-5a and α-SMA were from Novus. Antibody against active β-catenin was from Millipore. PCR reagents were purchased from Applied Biosystems. Bromodeoxyuridine (BrdU) cell proliferation assay, RNA isolation and complementary DNA (cDNA) synthesis kits were purchased from Millipore, Life Technology and Roche, respectively.

### Cell culture and treatments

The human lung fibroblasts (MRC5s) and the human alveolar epithelial adenocarcinoma cell line (A549) were obtained from American Type Culture Collection. Culture mediums were F-12 nutrient mixture and Dulbecco’s Modified Eagle’s Medium containing F^− 12^ for MRC5 and A549 cells, respectively. The cells of both cell lines were cultured in culture mediums supplemented with 100 U/mL penicillin, 100 μg/mL streptomycin, and 10% foetal bovine serum (FBS). The cells were grown in culture plates, at 37 °C in a humidified CO_2_ incubator. The cultured cells of both cell lines were treated with various concentrations of GRP (10^− 5^,10^− 6^ and 10^− 7^ M) for 24, 48 and 72 h (h). The cells were harvested at the end of the experiment. Three replicate experiments were evaluated for each analysis.

### Determination of cell viability

The MTT assay was used to assess cell viability. Briefly, cells were seeded in a 96-well flat-bottom microplate at a density of 1 × 10^4^ cells/well and allowed to adhere for 24 h at 37 °C in a CO_2_ incubator. The cultured cells were stimulated with 10^− 5^, 10^− 6^ and 10^− 7^ M concentrations of GRP for 24, 48 and 72 h. The culture medium was aspirated, and the cells were washed with PBS. A 30-μl volume of MTT working solution (5 mg/ ml in phosphate buffer solution) was added to each well and incubated for 4 h at 37 °C in a CO_2_ incubator. The medium was aspirated. Then, the formed formazan crystals were solubilized by adding 100 μL of DMSO per well. The intensity of the dissolved formazan crystals (purple color) was recorded at 570 nm by using a microplate reader (BioTek μQuant). Cell viability percentages were calculated by the formula: (mean optical density (OD) of treated cells /mean OD of control cells) × 100.

### Cell proliferation assay

BrdU cell proliferation assay kit (Millipore) was used to determine the proliferative response of cells. For the assay, MRC5 and A459 cells were plated at 1 × 10^4^ cells in 100 μL/well with appropriate cell culture media onto a 96-well tissue culture plate. The cells were treated with 10^− 5^, 10^− 6^ and 10^− 7^ M of GRP for 24, 48 and 72 h. BrdU (1X in 20 μl volume) was added to each well in the last 2 h of the GRP treatment. The amount of BrdU in cultured cells was measured using a kit according to the manufacturer’s instructions. Plates were read at 450 nm excitation and 550 nm emission wavelength using a microplate reader (BioTek μQuant).

### Immunoblotting

A549 (4 × 10^5^ cells per well) and MRC5 (4 × 10^5^ cells per well) cells were cultured onto 6-well plates. They were harvested at the end of the experiments and lyzed in RIPA buffer containing protease and phosphatase inhibitor cocktail. The protein concentration was quantified by the Bradford method. The protein samples (40 μg) were electrophoresed on 4–10% SDS-PAGE gels and blotted onto a nitrocellulose membrane. After 5% non-fat dry milk in tris-buffered saline containing Tween-20 (TBST) for 1 h at room temperature, the membrane was incubated with primary antibodies at 4 °C overnight. Following incubation, the membranes were washed in TBST buffer, incubated with labelled peroxidase secondary antibody goat anti-rabbit (Rb)-IgG or goat anti-mouse (Ms)-IgG for 1 h at room temperature and washed again. Then, they were incubated with enhanced chemiluminescence detection solution. Results were normalized to GAPDH. The optical density of bands was analyzed using Kodak Gel Logic Molecular Imaging Software (Kodak GL 1500, New Haven, CT, USA). Western blots are representative of three independent experiments. The following primary antibodies were used in 1:500 dilution: Rb anti-α-SMA, Ms. anti-fibronectin, Ms. anti-E-cadherin, Rb anti-Smad2/3, Rb anti-p-Smad2/3, Rb anti-Smad4, Rb anti-Smad7, Rb anti total β-catenin, Ms. anti-active β-catenin, Rb anti-Dkk1, Rb anti-Wnt4, Rb anti-Wnt5a, Rb anti-Wnt7a, Rb anti-GAPDH.

### Quantitative real time-PCR (QRT-PCR)

Total RNA was extracted from the cultured MRC5 cells (2 × 10^5^ cells per well) cultured onto 6-well plate using an RNA isolation kit (Invitrogen) according to the manufacturer’s instructions. cDNA was generated using the cDNA synthesis kit (Roche). A qRT-PCR method based on SYBR Green detection was carried out for gene expression quantification using the primers listed in Table 1. Real-time-PCR and data collection were performed with ABI StepOne Plus equipment. The housekeeping gene hypoxanthine-guanine phosphoribosyltransferase (*HPRT*) was used as an internal control to normalize the expression levels of different genes. The relative complementary DNA ratio was calculated using the value of threshold cycles (Ct). The amplification efficiency between the target and the reference control *HPRT* genes was compared by using the 2(−Delta Delta C(T), 2^–ΔΔCt^) method.

**Table 1:** Primers used for real-time PCR

Gene	Primer sequences (5′ → 3′)	
***ACTA2***	*Forward*	CGAGATCTCACTGACTACCTCATGA
	*Reverse*	AGAGCTACATAACACAGTTTCTCCTTGA
***VIM***	*Forward*	GGACCAGCTAACCAACGACA
	*Reverse*	AAGGTCAAGACGTGCCAGAG
***COL1A1***	*Forward*	CAAGAGGAAGGCCAAGTCGAG
	*Reverse*	TTGTCGCAGACGCAGATCC
***TGFB1***	*Forward*	CGTGCTAATGGTGGAAACCC
	*Reverse*	GGTAGTGAACCCGTTGATGT
***SMAD2***	*Forward*	CCAGGTCTCTTGATGGTCGT
	*Reverse*	TGGAGGCAAAACTGGTGTCT
***SMAD3***	*Forward*	GCATGGACGCAGGTTCTCC
	*Reverse*	GGCTCGCAGTAGGTAACTGG
***SMAD4***	*Forward*	GGAGGTGGCCTGATCTTCAC
	*Reverse*	CTTGGTGGATGCTGGATGGT
***SMAD7***	*Forward*	CGGAAGTCAAGAGGCTGTGT
	*Reverse*	TGGACAGTCTGCAGTTGGTT
***CTNNB1***	*Forward*	AAGTGGGTGGTATAGAGGCTCTTG
	*Reverse*	GATGGCAGGCTCAGTGATGTC
***DKK1***	*Forward*	GCACCCAGGCTCTGCAGTCA
	*Reverse*	GCACGGGTACGGCTGGTAGT
***GSK3B***	*Forward*	CTCATGCTCGGATTCAAGCA
	*Reverse*	GGTCTGTCCACGGTCTCCAGTA
***LEF1***	*Forward*	CATCAGGTACAGGTCCAAGAATGA
	*Reverse*	GTCGCTGCCTTGGCTTTG
***AXIN***	*Forward*	GAAGCGCGTGCGCATGGAGGA
	*Reverse*	GGCGGGAGGCAGCTTGTGAC
***GRP***	*Forward*	AGGTTCAAAAGGCAAAGGTTCT
	*Reverse*	GCAGAACGCAGTCTCTTAGG
***GRPR***	*Forward*	TGGCTAGACAGGAACCCTTG
	*Reverse*	CCTACACCACTCAGGAGCAT
***HPRT***	*Forward*	AAGGACCCCACGAAGTGTTG
	*Reverse*	GGCTTTGTATTTTGCTTTTCCA

### Immunofluorescence

The cultured MRC5 cells were seeded at 6 × 10^4^ per well onto 35 mm cell imaging dishes and they were incubated for 24 h at 37 °C in 5% CO_2_. The cells were treated with 10^− 5^ M of GRP for 24, 48 and 72 h. Cultured cells were fixed in a cold acetone/methanol mixture. After blocking with 5% BSA, cells were incubated with primary antibodies including mouse anti-α-SMA diluted 1:100 and rabbit anti-collagen1 diluted 1:200 at 4 °C overnight. Then, they were incubated with secondary antibodies including goat anti-mouse IgG conjugated with FITC and goat anti-rabbit IgG conjugated with Cy3 for 1 h at room temperature. The nuclei of cells were counterstained with 4′,6-diamidino-2-phenylindole (DAPI). Later on, cells were post-fixed with 4% paraformaldehyde for 10 min. Cells were visualized with a fluorescence microscope (Nikon, EclipseT*i*). Images were taken using a 40× oil-immersion objective.

### Statistical analysis

The GraphPad Prism 7.0 software program was used to evaluate statistically cell viability, BrdU cell proliferation, Western blotting and gene expression analysis in MRC5 and A549 cells treated with GRP. The mean, standard deviation and comparisons between the groups were bidirectionally performed by One-way ANOVA (using Bonferroni’s correction for multiple comparisons) and *Student t-test*. Results were reported as mean ± SE. *P* values less than 0.05 were considered significant.

## Results

### Cell viabilities of A549 and MRC5 cells treated with GRP

**Fig. 1:**
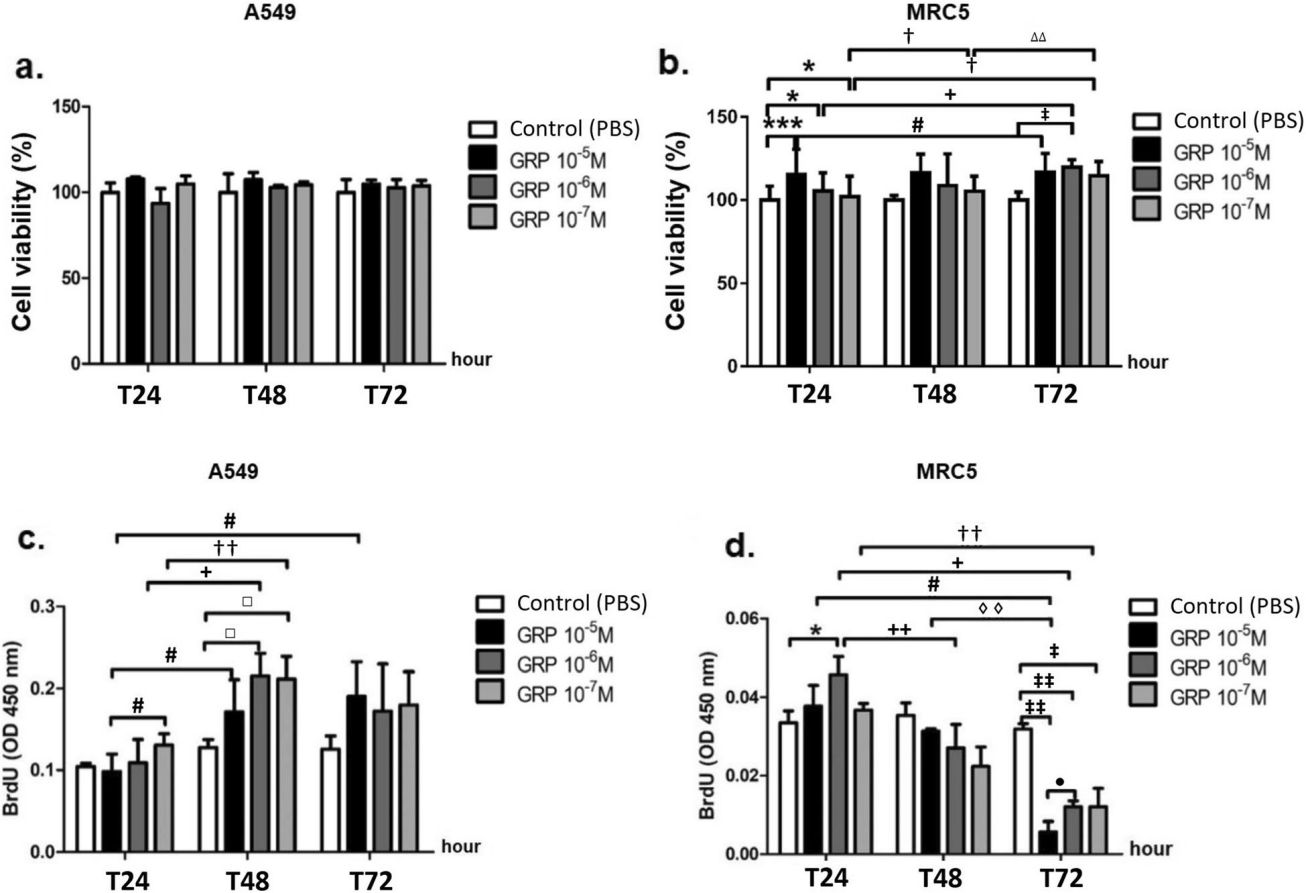
The effects of GRP on the cell viability (**a**, **b**) and the cell proliferation (**c**, **d**) of A549 and MRC5 cells. *P* values are *p < 0.05, **p < 0.01 and ***p < 0.001 compared to Control (PBS) at 24 h; ^**#**^*p* < 0.05 compared to GRP 10^− 5^ M at 24 h; ^**+**^*p* < 0.05 and ^**++**^*p* < 0.01 compared to GRP 10^− 6^ M at 24 h; ^†^*p* < 0.05 and ^††^*p* < 0.01 compared to GRP 10^− 7^ M at 24 h; ^□^*p* < 0.05 compared to Control (PBS) at 48 h; ^◊◊^*p* < 0.01 compared to GRP 10^− 5^ M at 48 h; ^∆∆^*p* < 0.01 compared to GRP 10^− 7^ M at 48 h; ^‡^*p* < 0.05 and ^‡‡^*p* < 0.01 compared to Control (PBS) at 72 h; ^•^*p* < 0.05 compared to GRP 10^− 5^ M at 72 h

GRP (10^− 5^, 10^− 6^ and 10^− 7^ M) did not reduce the viability of A549 and all doses of it increased the viability of MRC5 cells. The percentage of the cell viability was approximately ≥90% for both cell lines at 24, 48 and 72 h following stimulation, when compared with the baseline (PBS) (Fig. 1a, b, Table S1).

### Proliferative responses of A549 and MRC5 cells treated with GRP

In A549 cells stimulated with a 10^− 5^ M dose of GRP, BrdU level was not altered at 24 h, 48 h and 72 h compared with their controls. The 10^− 6^ and 10^− 7^ M doses of GRP significantly increased BrdU levels in A549 cells at 48 h compared with the Control at 48 h (*p* < 0.05). The 10^− 6^ and 10^− 7^ M doses of GRP stimulated the proliferation of A549 cells at 48 h (Fig. 1c, Table S1).

All doses of GRP (10^− 5^, 10^− 6^ and 10^− 7^ M) increased BrdU levels in MRC5 cells in the first 24 h. The increase at 10^− 6^ M was significant compared with Control at 24 h (*p* < 0.05). Each of the three doses reduced BrdU levels of fibroblasts at 48 h and, especially, 72 h compared with their controls (*p* < 0.01 for 10^− 5^ and 10^− 6^ M of GRP; *p* < 0.05 for 10^− 7^ M of GRP). The 10^− 5^ and 10^− 6^ M doses of GRP were the most effective in reducing cell proliferation (Fig. 1d, Table S1).

### GRP does not induce epithelial-mesenchymal transition in A549 cells

**Fig. 2:**
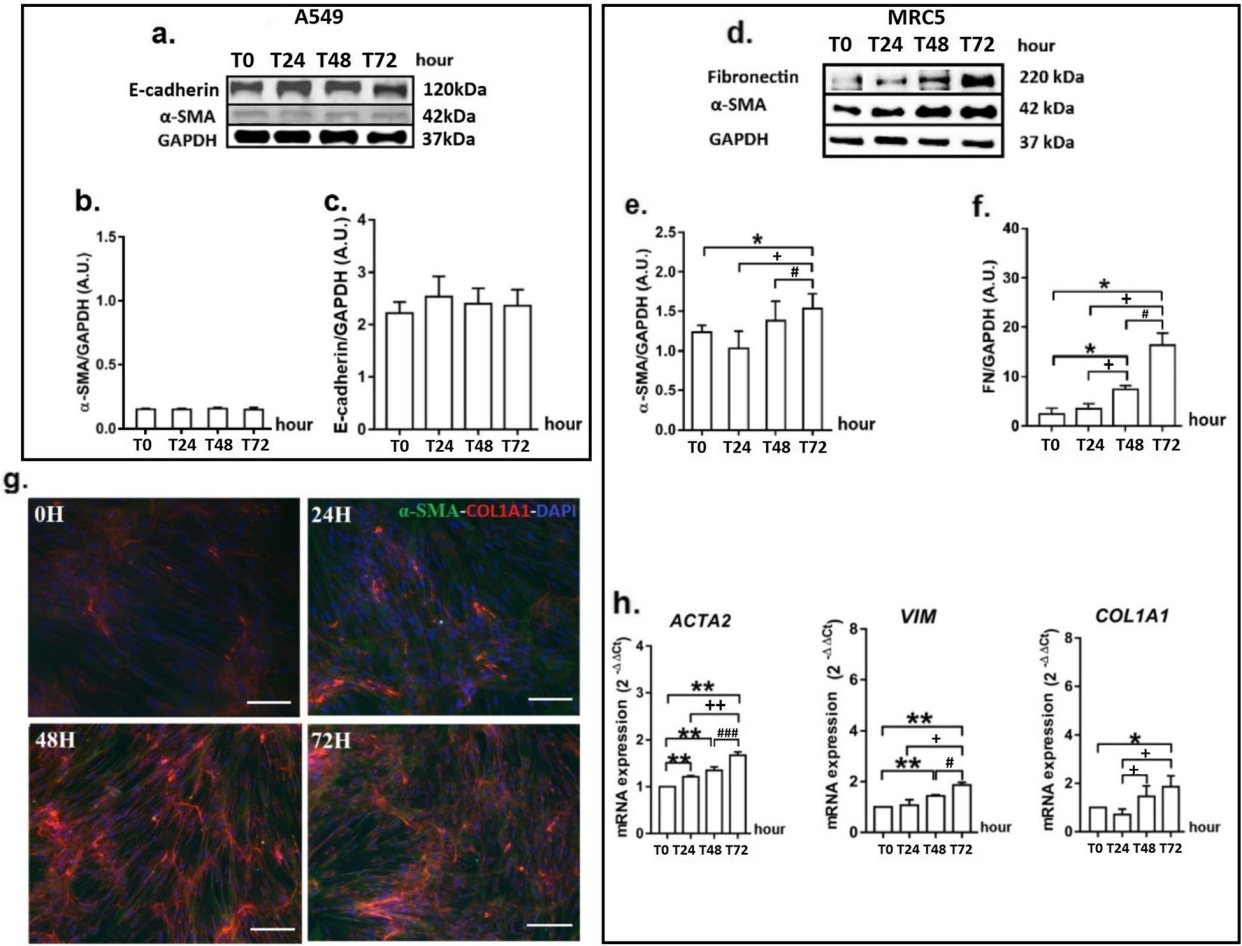
GRP does not induce epithelial-mesenchymal transition in A549 cells. Figure shows protein bands of α-SMA and E-cadherin (**a**), and the alterations at the protein levels of α-SMA (**b**) and E-cadherin (**c**). GRP induces the differentiation into myofibroblasts and the production of ECM components in MRC5. Figure shows protein bands of α-SMA and Fibronectin (**d**), the alterations at protein levels of α-SMA (**e**) and fibronectin (FN) (**f**), the immunoreactivity at 0-72 h periods of α-SMA (green) and Collagen 1α1 (red) (**g**), and the changes at gene expression of *ACTA2*, *VIM*, *COL1A1*(h). *P* values are *p < 0.05 and **p < 0.01 compared to Time (0 h); ^+^*p* < 0.05 and ^++^*p* < 0.01 compared to Time (24 h); ^#^*p* < 0.05 and ^###^*p* < 0.001 compared to Time (48 h). Original magnification ×400; scale bar, 50 μm

The 10^− 5^ M dose of GRP did not change the protein levels of E-cadherin (epithelial marker) and α-SMA (myofibroblast marker) at 24, 48 and 72 h compared with 0 h in A549 cells (Fig. 2a, b).

### GRP stimulates the differentiation of fibroblasts into myofibroblasts and the production of ECM components

In MRC5 fibroblast cells stimulated with 10^− 5^ M dose of GRP, an increase in the amount of α-SMA protein was observed from 48 h of experiment, that became significant at 72 h compared with 0 h (*p* < 0.05, Fig. 2d, e). The fibronectin protein level was significantly increased by GRP at 48 h (*p* < 0.05) and peaked at 72 h (*p* < 0.05) (Fig. 2d, f). Additionally, immunofluorescence labelling revealed the increased collagen-1 and α-SMA immunoreactivities at 48 h following stimulation of GRP (Fig. 2g). These findings were confirmed by qRT-PCR analysis of *ACTA2, VIM* and *COL1A1* genes. GRP induced expressions of *ACTA2*, *VIM* and *COL1A1* genes at 24 and 48 h of stimulation, respectively, as time progressed (Fig. 2h).

### GRP stimulates myofibroblast differentiation by inducing Smad dependent-TGF-β signalling in MRC5 cells

**Fig. 3:**
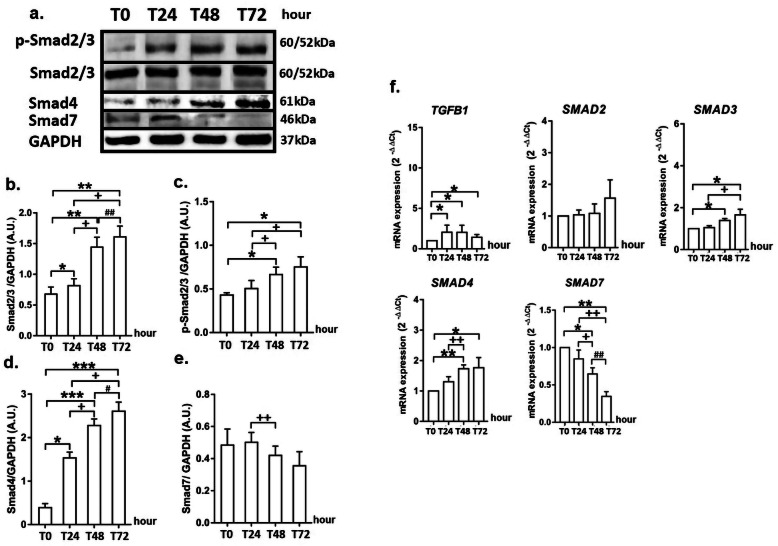
GRP stimulates myofibroblast differentiation by inducing Smad dependent-TGF-β signalling in MRC5 cells. Figure shows the protein bands of phospho-Smad2/3, Smad2/3, Smad4, Smad7 (**a**), the changes at protein levels of Smad2/3 (**b**), p-Smad2/3 (**c**), Smad4 (**d**) and Smad7 (**e**), and the alterations at gene expression of *TGFB1*, *SMAD2*, *SMAD3*, *SMAD4* and *SMAD7* (**f**). P values are *p < 0.05, **p < 0.01 and ***p < 0.001 compared to Time (0 h); ^+^*p* < 0.05 and ^++^*p* < 0.01 compared to Time (24 h); ^#^*p* < 0.05 and ^##^*p* < 0.01 compared to Time (48 h)

The stimulation of MRC5 cells with GRP 10^− 5^ M caused a significant increase at the protein levels of total-Smad2/3 and Smad4 at 24 h (*p* < 0.05 for total-Smad2/3 and Smad4), the protein levels of p-Smad2/3 as well as total Smad2/3 and Smad4 at 48 and 72 h (*p* < 0.01 for total-Smad2/3 and *p* < 0.001 for Smad4) compared with 0 h (*p* < 0.05) (Fig. 3a, d). The protein levels of Smad7 did not alter at 48 h of the experiment, compared with 0 h (Fig. 3a, e).

In the MRC5 cells stimulated with GRP, *TGFB1* gene expression was progressively increased at 24, 48 and 72 h when compared with 0 h (*p* < 0.05). The expression levels of the *SMAD2* gene after GRP stimulation did not alter (Fig. 3f). GRP stimulation increased significantly the expression levels of *SMAD3* and *SMAD4* genes at 48 and 72 h compared with 0 h (*p* < 0.05 for *SMAD3* gene; *p* < 0.05 and *p* < 0.01 for *SMAD4* gene). GRP administration decreased *SMAD7* gene expression at 24 and 48 h (*p* < 0.05) and reached its highest level at 72 h (*p* < 0.01) compared with 0 h (Fig. 3f).

### GRP induces myofibroblast differentiation by inducing Wnt signalling in MRC5 cells

**Fig. 4:**
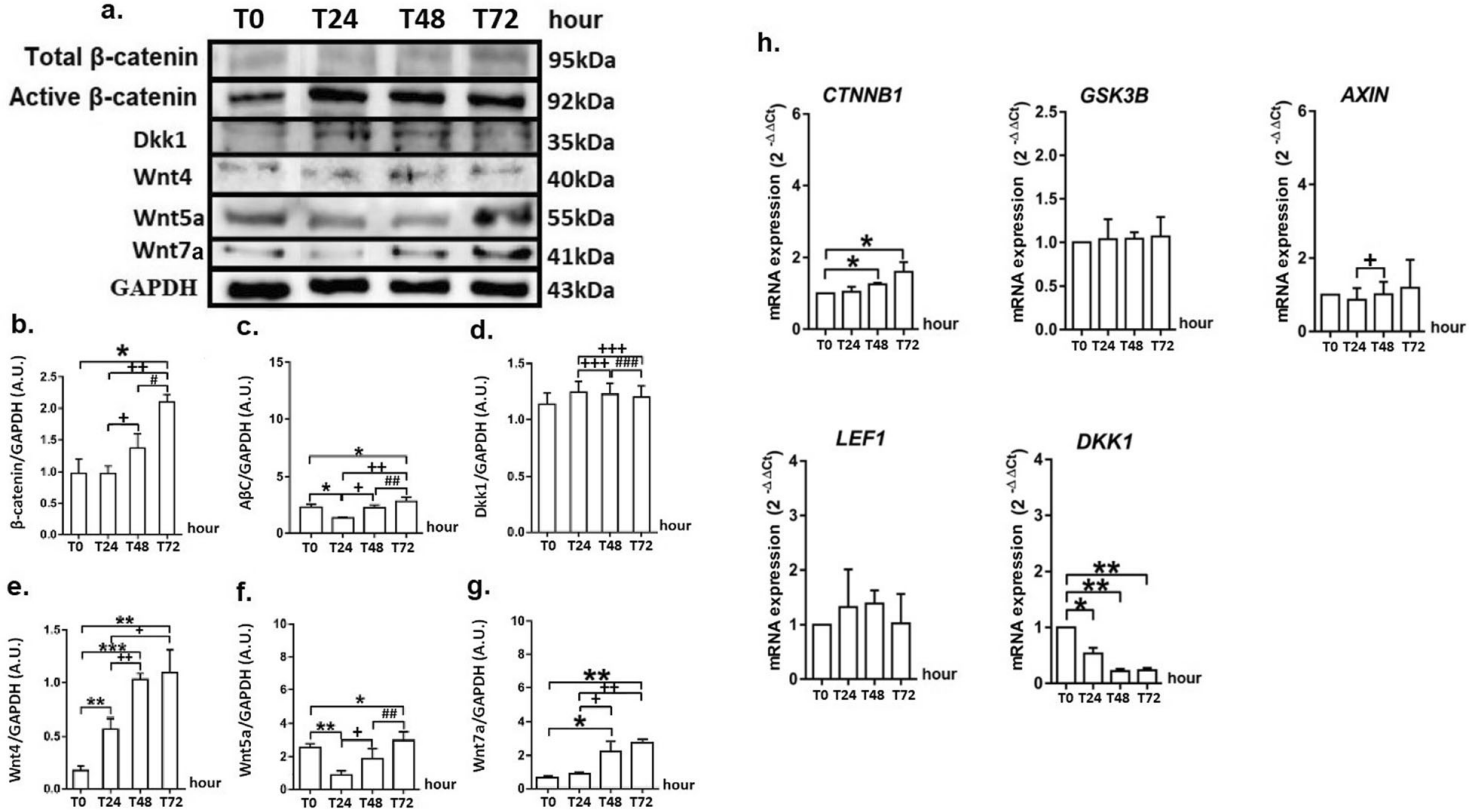
GRP induces myofibroblast differentiation by inducing Wnt signalling in MRC5 cells. Figure shows the protein bands of total β-catenin, non-phospho (active) β-catenin, Dkk1, Wnt4, Wnt5a, and Wnt7a (**a**), the changes at protein levels of total β-catenin (**b**), active β-catenin (**c**), Dkk1 (**d**), Wnt4 (**e**), Wnt5a (**f**) and Wnt 7a (**g**), and the alterations at the gene expression of *CTNNB1*, *GSK3B*, *AXIN*, *LEF1*, and *DKK1* (**h**). P values are *p < 0.05, **p < 0.01 and ***p < 0.001 compared to Time (0 h); ^+^*p* < 0.05, ^++^*p* < 0.01 and ^+++^*p* < 0.001 compared to Time (24 h); ^#^*p* < 0.05, ^##^*p* < 0.01 and ^###^*p* < 0.001 compared to Time (48 h)

GRP increased significantly the protein levels of total β-catenin and active β-catenin at 72 h compared with the protein levels determined at 0, 24 and 48 h (*p* < 0.05, Fig. 4a-c) in MRC5 cells. GRP stimulation did not cause a change in Dkk1 levels in the same cell line (Fig. 4a, d). Wnt4 protein levels increased following GRP stimulation, at 24 (*p* < 0.01), 48 (*p* < 0.001) and 72 (*p* < 0.01) h in MRC5 cells (Fig. 4a, e). The level of Wnt5a decreased in the first 24 h following GRP stimulation (*p* < 0.01) and increased significantly at 72 h of the experiment (*p* < 0.05, Fig. 4a, f). GRP increased Wnt7a level in MRC5 cells at 24 h following stimulation, especially at 48 (*p* < 0.05) and 72 h (*p* < 0.01, Fig. 4a, g).

*CTNNB1* gene expression increased significantly in the MRC5 cells treated with GRP at 48 h compared with 0 h (*p* < 0.05) and reached a peak at 72 h (*p* < 0.05). GRP stimulation did not alter the expression of *GSK3B*, *AXIN1* and *LEF1* genes at 24, 48 and 72 h compared with 0 h in MRC5 cells. GRP reduced *DKK1* gene expression at 24 (*p* < 0.05),48 (*p* < 0.01) and 72 (*p* < 0.01) h compared with 0 h in MRC5 cells (Fig. 4h).

### GRP is effective on MRC5 cells via autocrine and paracrine signallings

**Fig. 5:**
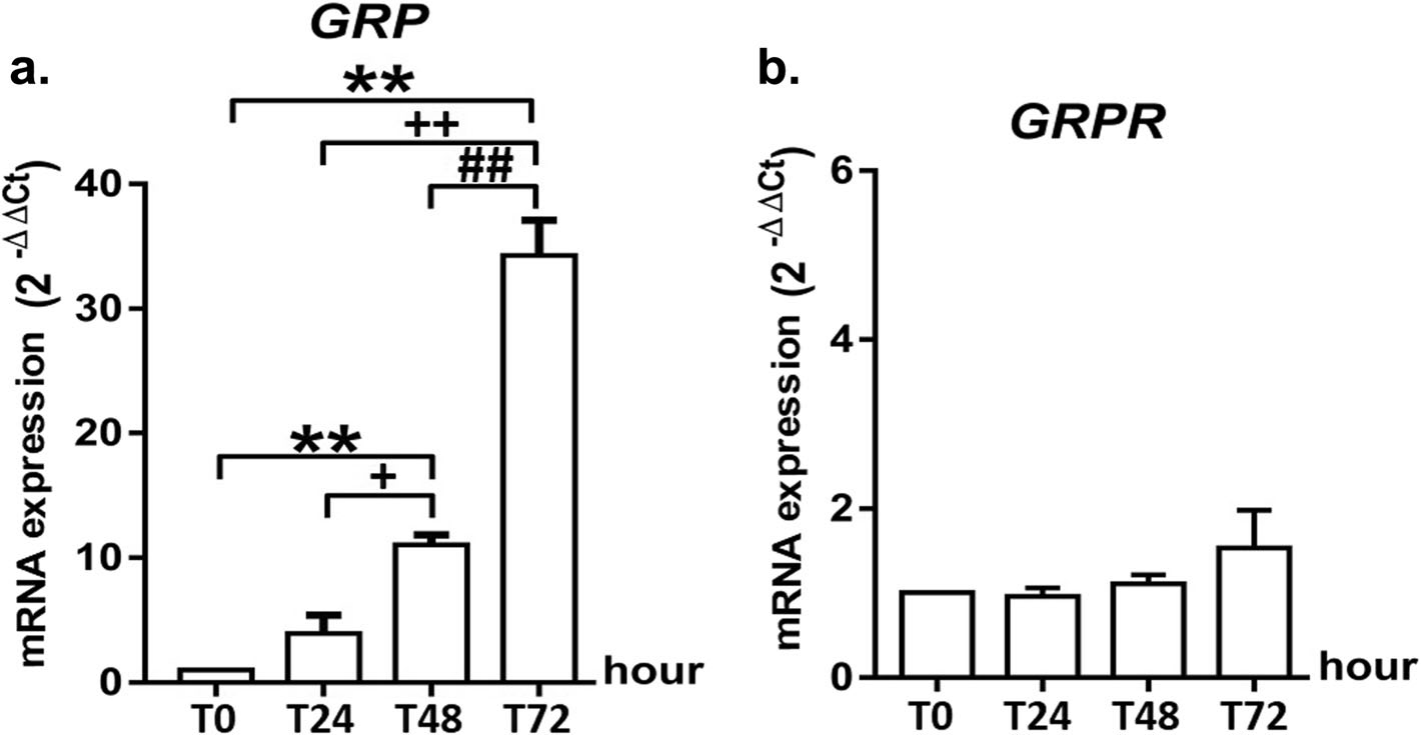
GRP is effective on MRC5 cells via autocrine and paracrine signalling. Figure shows the change at gene expression of *GRP* (**a**) and *GRPR* (**b**) after GRP treatment in MRC5 cells P values are **p < 0.01 compared to Time (0 h); ^+^*p* < 0.05 and ^++^*p* < 0.01 compared to Time (24 h); ^##^*p* < 0.01 compared to Time (48 h)

Although GRP induced the expression of *GRP* gene at 48 h compared to 0 h (*p* < 0,01) and 24 h (*p* < 0,05) and 72 h compared with 0 h (*p* < 0,01), 24 h (*p* < 0,01) and 48 h (*p* < 0,01) in MRC5 cells (Fig. 5a), it did not cause a change in expression of its receptor gene (*GRPR*) (Fig. 5b).

## Discussion

The present study provided the first data regarding the crosstalk between GRP, TGF-β and Wnt signalling pathways and their active role in the differentiation of MRC5 cells into myofibroblasts and in the production of ECM elements. Furthermore, we found that human lung fibroblasts are the major target of GRP acting on target cells via autocrine and paracrine signalling. GRP also contributes to the pathogenesis of pulmonary fibrosis by stimulating proliferation of MRC5 fibroblast early on and their differentiation into myofibroblasts in the later period. GRP does not induce EMT in A549 cells.

Fibroblast activation induces the formation of pulmonary fibrosis. Fibroblasts proliferate in the first step of fibroblast activation. In the second step, fibroblasts differentiate into myofibroblasts. Fibroblasts accumulate in fibrotic foci of fibrotic lungs. Yilmaz et al. observed that the number of Ki-67^+^ cells (proliferative cells) increased in fibrotic foci in the murine lungs with pulmonary fibrosis induced by bleomycin [18]. It is known that lung fibroblasts of patients with idiopathic pulmonary fibrosis (IPF) have a higher proliferative capacity than healthy lung fibroblasts [19]. Fibroblasts isolated from IPF patients were induced to proliferate by PI3K-Akt signalling [20] and showed increased Wnt5a expression [21]. GRP is produced by PNECs in the epithelium covering the airways of the human lungs. In a study carried out on newborn mice (aged 1–3 postnatal days) treated with exogenous bombesin/GRP and sacrificed on day 14 after the treatment, proliferation, thickening of the alveolar wall and bronchodysplasia was detected in the mice lung [15]. BrdU method labels cells in the S phase. The increased amount of BrdU correlates with the increased number of proliferating cells in S phase [22]. The data of the present study show for the first time that GRP may be effective in stimulating the proliferation of human lung fibroblasts in the early period (the first 24 h after stimulation), and reducing cell proliferation at the later stages at 48 h and 72 h.

GRP immunoreactivity was found in the hyperplastic foci of the bronchioles in the human fibrotic lungs [23]. The release of bombesin-like peptide from alveolar macrophages was determined in BAL fluid extracted from the fibrotic lungs of rats treated with lipopolysaccharide [24]. In another study, administration of bombesin/GRP to mice in the first 3 postnatal days increased α-SMA immunoreactivity in the lungs, caused a significant increase of the alveolar wall thickness and decreased alveolarization on the 14th day after birth. All these effects were lost in the *GRPR* gene knockout mice [15]. In another study, hyperplasia of PNECs, increased nuclear p-Smad2/3 immunoreactivity, induced α-SMA expression and interstitial collagen accumulation were observed in the 6th, 10th and 20th weeks following the administration of high-dose of thoracic radiation to murine lungs. The administrations of GRP antagonist to mice (1 h after radiation exposure on the 20th week) resulted in the regression of these alterations [16]. Although this last study showed that GRP might modulate the pathogenesis of pulmonary fibrosis, the target cells and signalling pathways of GRP-mediated generation of fibrotic response were not investigated. In the present study, the direct effect of GRP on human lung fibroblasts was tested for the first time. In addition, we found that GRP treatment of MRC5 fibroblasts induces myofibroblast differentiation by increasing α-SMA protein and gene expression at 48 h and especially, 72 h of stimulation. Such results indicate clearly that human lung fibroblasts are target cells of GRP, and that GRP is effective in stimulating the synthesis of collagen and fibronectin proteins in these cells in addition to the induction of the differentiation of MRC5 fibroblasts into myofibroblasts. When a cell is differentiated, its proliferation stops. In the present study, MRC5 fibroblast cells stimulated by GRP showed a significantly reduced proliferation at 48 h and 72 h following stimulation compared to 0 and 24 h. They also increased the synthesis of α-SMA, collagen and fibronectin proteins and interstitial collagen accumulation, while decreasing the cell proliferation. Based on these findings, we can say that GRP may stimulate the proliferation of MRC5 fibroblasts in the early period following stimulation, but in the later period it induces the differentiation of these cells into myofibroblasts, thereby enabling the production of ECM elements such as collagen and fibronectin.

EMT allows the alveolar epithelial cells to differentiate into myofibroblasts, resulting in an increased number of myofibroblasts and production of ECM components by these cells. Kim et al. reported weak pro-SPC and N-cadherin immunoreactive cells in the interstitium of patients with IPF [25]. In addition, the increased expressions of mesenchymal cell proteins such as α-SMA, collagen and calponin1 were detected in epithelial cells isolated from fibrotic areas in patients with IPF [26]. Bleomycin treatments on A549 cells for 4 days reduced the expression of E-cadherin by stimulating the TGF-β/Smad3 signalling pathway, while it induced α-SMA and vimentin expressions. A549 cells differentiate into myofibroblasts by EMT activation [27]. Many studies report that when A549 cells are stimulated with TGF-β, the cells begin to produce mesenchymal cell proteins such as vimentin, desmin, N-cadherin, α-SMA, fibronectin and collagen instead of epithelial cell markers such as E-cadherin, cytokeratin and zonula okludens-1. In other words, they convert their epithelial phenotype into mesenchymal phenotype [25, 28, 29]. In the present study, GRP did not stimulate the differentiation of A549 cells into myofibroblasts via EMT, whereas it was able to differentiate MRC5 fibroblast into myofibroblasts. Therefore, we suggest that the alveolar epithelium is not a target for GRP in order to get a fibrotic response in the lung via EMT. Jaeger and colleagues reported that GRP had no proliferative effect on A549 cells on 24 h after GRP (50 and 100 nM) administration, but it stimulated ROS production and cell migration of these cells [30]. However, it has been shown for the first time that GRP application causes the proliferation of A549 cells in the present study. We think that GRP may stimulate the proliferation of A549 cells as opposed to its incapacity to regulate the differentiation of A549 cells into myofibroblast via EMT.

Adenovector-mediated gene transfer of active TGF-β1 resulted in increased endogenous TGF-β1 expression in rat lungs within a few days after administration of the vector, and this application induced pulmonary fibrosis without any inflammation in the rat lungs. Moreover, the formation of fibrotic foci and myofibroblast differentiation were detected, as well [31]. In another study, it was shown that mice could be protected against pulmonary fibrosis when TGF-β signal was inhibited [32]. TGF-β modulates ECM turnover by stimulating the production of ECM components such as collagen and fibronectin and controlling the activities of MMPs and tissue metalloproteinases [33, 34]. The most important contribution of TGF-β signalling to the pathogenesis of pulmonary fibrosis is the stimulation of myofibroblast differentiation. The increased TGF-β1 signalling in the lungs enables the differentiation of various cell types, particularly fibroblasts and type 2 pneumocytes, into myofibroblasts [29, 35]. Unlike normal lungs, TGF-β1 expression is increased in lung tissue of IPF patients [36]. Activation of the Smad-dependent TGF-β1 signalling is correlated with increases at the levels of Smad2/3 and Smad4, activation of Smad2/3 proteins and the decrease of the level of Smad7 (inhibitor of TGF-β signalling) in the cell and tissue [37]. It has been found that activation of the transcriptional complex of Smad3 is required to induce the expression of collagen-1 genes in human skin fibroblasts [38]. Increased levels of p-Smad3 were detected in the fibrotic lungs of the mice treated with bleomycin, and in mouse fibroblasts treated with TGF-β [39]. The loss of Smad3 in the mutant mice treated with bleomycin resulted in the reduction of the procollagen-1 mRNA expression induced by TGF-β and the decrease in the amount of hydroxyproline in the lung [40]. The stimulation of nuclear localization of Smad4 protein mediated by *α*B-crystalline in primary fibroblast cells isolated from fibrotic mouse lungs increased the expression of TGF-β1 target genes, such as α-SMA and procollagen-1 genes in these cells [41]. After adenovector-mediated Smad7 gene transfer in fibrotic mice lung, stimulation of Smad7 mRNA expression caused the reduction of pulmonary fibrosis in the mice [42]. These results demonstrate that the phosphorylation of Smad2/3 proteins, transfer of Smad4 protein to the nucleus and reduction of the amount of Smad7 inhibitor protein are needed for the activation of the Smad-dependent TGF-β signal pathway. These alterations in the pathway resulted in a fibrotic response in the lung, by increasing the expression of α-SMA and procollagen-1 genes. Although there is no direct evidence that GRP stimulates TGF-β signalling pathway, Zhou and colleagues showed that the radiation induced fibrosis by increasing the number of p-Smad2/3 immunoreactive cells in the lungs of mice and the administration of GRP inhibitor regressed the pulmonary fibrosis by decreasing the number of p-Smad2/3 in the lungs [16]. The present study reveals that GRP stimulates Smad3-dependent TGF-β1 signalling, by increasing the expressions of SMAD3 and SMAD4 mRNAs, total Smad2/3, p-Smad2/3 and Smad4 proteins and reducing the expression of SMAD7 mRNA in MRC5 cells. Thus, here we show that GRP stimulates the differentiation of fibroblasts into myofibroblasts via Smad3-dependent TGF-β signalling pathway.

Wnt/β-catenin signalling plays an important role in the pathogenesis of pulmonary fibrosis. WNT1-inducible-signalling pathway protein 1 (WISP1), the secreted frizzled-related protein 2 (sFRP2), and active β-catenin increased in IPF causing an over-activation of Wnt signalling pathway [43–46]. Akhmetshina and colleagues found that canonical Wnt signalling was required for TGF-β-mediated fibrosis in fibroblasts and mouse lungs [47]. In addition, the authors demonstrated that the excessive expression of Dkk1 prevented pulmonary fibrosis in transgenic mice and that the stimulation of Wnt signalling was induced when fibroblasts, induced by TGF-β, decreased Dkk1 expression. In another study, TGF-β administration to human IMR-90 fibroblasts resulted in the induction of Wnt activation [48]. There is no study on the relation between GRP and Wnt signalling. The present study contains some evidences that GRP may activate Wnt signalling together with TGF-β signalling in MRC5 cells. GRP increased active-β catenin and total β-catenin protein levels, β-catenin mRNA expression and decreased Dkk1 mRNA expression in MRC5 cells. In contrast, GRP had no effect on GSK-3β, AXIN and LEF1 mRNA levels. We suggest that GRP stimulates the differentiation of fibroblasts into myofibroblast by β-catenin activation in MRC5 fibroblasts. These results are important because they are the first data to show that GRP might be effective in stimulating Wnt signalling.

Increased level of active β-catenin protein and expressions of Wnt3a, Wnt4 and Wnt7a mRNA accompanied pulmonary fibrotic response to bleomycin treatment in mice lungs [49]. In another study, the addition of exogenous Wnt4 and Wnt5a to the culture medium of normal human lung fibroblasts and 3D human lung tissue resulted in an increased level of S100A4 mRNA, a marker of the myofibroblast. Therefore, it has been suggested that these Wnt ligands may play a role in myofibroblast differentiation [50]. This suggestion was confirmed by another study. Wnt5a contributes to pulmonary fibrosis by stimulating the fibroblast proliferation in human lung with usual interstitial pneumonia [21]. In primary fibroblasts isolated from human lungs with IPF, Wnt5a stimulated the proliferation of pulmonary fibroblasts [51]. It can be produced by many cells of the lung with IPF, and its expression is increased by TGF-β1 and Wnt7b [52]. FZD7-Wnt5a interaction stimulates the production of ECM components via non-canonical Wnt signalling [53]. Additionally, increased Wnt4 expression has been reported in the wound repair in foetal lung fibroblasts after TGF-β stimulation [54]. Moreover, the presence of Wnt7b in fibroblastic foci in the lungs of patients with IPF was determined immunohistochemically [55]. Gene expression analysis showed that the amount of Wnt7b mRNA was increased in these regions. Furthermore, it was found that the overexpression of Wnt7b was effective in increasing the production of procollagen in lung fibroblasts [56]. The results of the above studies show that Wnt4 and Wnt5a expressions can be induced by TGF-β. In addition, Wnt7b/β-catenin signalling may also stimulate Wnt5a expression. Wnt5a stimulates cell proliferation and ECM production in human lung fibroblasts. There are no data on the effect of Wnt7a on myofibroblast differentiation. The presence of Wnt7a was only detected in fibrotic lungs of mice. Studies describing the effects of GRP on Wnt ligands are still missing. In the present study, the levels of Wnt5a, Wnt7a and active β-catenin were low in MRC5 fibroblasts stimulated with GRP in the first 24 h, whereas the levels of these proteins increased significantly at 72 h of the experiment. The amount of Wnt4 protein increased steadily from 24 h to 72 h. Accordingly, we suggest that GRP may regulate the myofibroblast differentiation by Wnt4, Wnt5a and Wnt7a ligands in MRC5 cells. This was confirmed by TGF-β signalling and β-catenin activations on 72 h, when the myofibroblast differentiation and ECM production reached its peak. GRP stimulates the differentiation of fibroblasts into myofibroblasts and production of ECM by activation of TGF-β and Wnt signalling pathways. Based on the results of the present study, crosstalk between GRP, TGF-β and Wnt signalling pathways might play an active role in the differentiation of MRC5 cells to myofibroblasts and in the production of ECM elements.

One of the original findings of the present study is that GRP can affect MRC5 fibroblast cells by both autocrine and paracrine signalling. We demonstrated that GRP stimulates the expression of *GRP* genes in MRC5 fibroblast cells but is not effective in stimulating the gene expression of its receptor in these cells. This finding point outs that GRP acts on target cells via autocrine and paracrine signalling.

## Conclusion

Human lung fibroblasts are the major target cells of GRP during the generation of fibrotic responses. GRP contributes to the pathogenesis of pulmonary fibrosis by stimulating the proliferation of MRC5 fibroblast cells at an early stage and their differentiation into myofibroblasts in the later on. These effects are dose and time dependent. The results of the present study state for the first time, that the crosstalk between GRP, TGF-β and Wnt signalling pathways play an active role in the differentiation of MRC5 cells into myofibroblasts and in the production of ECM elements.

## Supplementary information

**Additional file 2: Table S1.** The effect of different concentrations of GRP on the viability and proliferation of human lung adenocarcinoma (A549) and human fetal lung fibroblast (MRC5) cells.

## Data Availability

All data generated or analysed during this study are included in this published article.

## Abbreviations

ECM: Extracellular matrix
IPF: Idiopathic pulmonary fibrosis
PNECs: Pulmonary neuroendocrine cells
GRP: Gastrin-releasing peptide
α-SMA: alpha-smooth muscle actin
TGF-β: Transforming growth factor-beta
MRC5: Human lung fibroblast cell line
A549: Adenocarcinomic human alveolar epithelial cell line
AEC2: Type 2 alveolar epithelial cells
EMT: Epithelial-mesenchymal transition
qPCR: quantitative polymerase chain reaction
MTT: 3-(4,5-dimethylthiazol-2-yl)-2,5-diphenyltetrazolium bromide
BSA: Bovine serum albumin
Dkk-1: Dickkopf-related protein 1
GAPDH: Glyceraldehyde 3-phosphate dehydrogenase
BrdU: Bromodeoxyuridine
cDNA: complementary DNA
HPRT: Hypoxanthine-guanine phosphoribosyltransferase
