# Supplementary material for: Gastrin-releasing peptide induces fibrotic response in MRC5s and proliferation in A549s
Source: Cell Commun Signal. 2020 Jun 18;18:96. doi: 10.1186/s12964-020-00585-y (PMC7301567; doi:10.1186/s12964-020-00585-y)
Supplement: Supplementary file 2 — Additional file 2: Table S1. The effect of different concentrations of GRP on the viability and proliferation of human lung adenocarcinoma (A549) and human fetal lung fibroblast (MRC5) cells. [file 12964_2020_585_MOESM2_ESM.docx]

**Supplementary Data 1**

**Table S1**. The effect of different concentrations of GRP on the viability and proliferation of human lung adenocarcinoma (A549) and human fetal lung fibroblast (MRC5) cells.

|  | | | A549 ^a^ | | MRC5 ^b^ | |
| --- | --- | --- | --- | --- | --- | --- |
| Time after  treatment (h) | GRP concentration  (M) | Viability (%) | | Proliferation  (OD 450nm) | Viability (%) | Proliferation  (OD 450nm) |
| T24 | Control (PBS) | 100±5.47 | | 0.104±0.004 | 100±8.30 | 0.033±0.003 |
|  | 10^-5^ | 107.67±1.23 | | 0.098±0.021 | 115.45±15.06**^***^** | 0.038±0.005 |
|  | 10^-6^ | 93.45±8.77 | | 0.109±0.029 | 105.49±10.87**^*^** | 0.046±0.005**^*^** |
|  | 10^-7^ | 104.87±4.69 | | 0.131±0.014**^#^** | 102.02±12.34**^*^** | 0.037±0.002 |
| T48 | Control (PBS) | 100±10.83 | | 0.128±0.010 | 100±2.72 | 0.035±0.003 |
|  | 10^-5^ | 107.34±4.21 | | 0.171±0.040**^#^** | 116.38±11.28 | 0.031±0.001 |
|  | 10^-6^ | 102.84±1.21 | | 0.215±0.027**^+/□^** | 108.70±19.12 | 0.027±0.006**^++^** |
|  | 10^-7^ | 104.3±1.59 | | 0.212±0.027**^††/□^** | 105.26±9.01**^†^** | 0.022±0.005 |
| T72 | Control (PBS) | 100±7.48 | | 0.126±0.016 | 100±4.84 | 0.032±0.001 |
|  | 10^-5^ | 104.81±2.37 | | 0.190±0.042**^#^** | 116.75±11.28**^#^** | 0.006±0.003**^#/◊◊/‡‡^** |
|  | 10^-6^ | 102.73±4.71 | | 0.172±0.058 | 119.80±4.54**^+/‡^** | 0.012±0.005**^+/‡‡/•^** |
|  | 10^-7^ | 103.67±3.31 | | 0.180±0.040 | 114.57±8.59^†/∆∆^ | 0.012±0.005^††/‡^ |

**^a^** Both cell line (1x10^4^ cells) were incubated in triplicate with the indicated concentrations (Molar (M)) of GRP for the indicated time and then examined for cell viability and proliferation by MTT and BrdU method, respectively. The results shown as mean of triplicate ± s.d. P values are *p<0.05, **p<0.01 and ***p<0.001 compared to Control (PBS) at 24h; **^#^**p<0.05 compared to GRP 10^-5^M at 24h; **^+^**p<0.05 and **^++^**p<0.01 compared to GRP 10^-6^M at 24h; ^†^p<0.05 and ^††^p<0.01 compared to GRP 10^-7^M at 24h; ^□^p<0.05 compared to Control (PBS) at 48h; ^◊◊^p<0.01 compared to GRP 10^-5^M at 48h; ^∆∆^p<0.01 compared to GRP 10^-7^M at 48h; ^‡^p<0.05 and ^‡‡^p<0.01 compared to Control (PBS) at 72h; ^•^p<0.05 compared to GRP 10^-5^M at 72h.
